# Development of a Computer Vision-Based Muscle Stimulation Method for Measuring Muscle Fatigue during Prolonged Low-Load Exposure

**DOI:** 10.3390/ijerph182111242

**Published:** 2021-10-26

**Authors:** Bochen Jia, Abhishek Nagesh Kumbhar, Yourui Tong

**Affiliations:** 1 Industrial and Manufacturing System Engineering, University of Michigan—Dearborn, Dearborn, MI 48128, USA; tongy@umich.edu; 2 Somnio Global, LLC., 45145 W 12 Mile Rd., Novi, MI 48377, USA; akumbhar@umich.edu

**Keywords:** muscle fatigue, computer vision, muscle stimulation, low-load exposure, ergonomics, prolonged exposure

## Abstract

Measuring muscle fatigue is one essential and standard method to quantify the ergonomic risks associated with prolonged low-load exposure. However, measuring muscle fatigue using EMG-based methods has shown conflicting results under low-load but sustained work conditions, e.g., prolonged sitting. Muscle stimulation technology provides an alternative way to estimate muscle fatigue development during such work conditions by monitoring the stimulation-evoked muscle responses, which, however, could be restricted by the accessibility and measurability of targeted muscles. This study proposes a computer vision-based method to overcome such potential restrictions by visually quantifying the muscle belly displacement caused by muscle stimulation. The results demonstrate the ability of the developed computer vision-based stimulation method to detect muscle fatigue from prolonged low-load tasks. Current results can be used as a foundation to develop a sensitive and reliable method to quantify the adverse effects of the daily low-load sustained condition in occupational and nonoccupational settings.

## 1. Introduction

The rapid developments of technology and changes in the nature of modern work have led to more seated work, which usually requires sustained low-level muscle contractions to maintain a seated working posture. For example, only ~3% maximum voluntary contraction (MVC) of trunk extensor muscles [1,2] and less than 7% MVC of the forearm, shoulder, and neck extensor muscles have been reported during various seated tasks [3,4]. Although the levels of muscle contractions are low (i.e., <10% MVC), both objective and subjective signs of fatigue were observed for medium to long exposure (e.g., >40 min) [5,6,7,8,9,10,11].

The presence of muscle fatigue is one important indicator to quantify the potential risks associated with sedentary work and lifestyle on the musculoskeletal system. Measuring muscle electromyography (EMG) is a common method to quantify the development of muscle fatigue. However, measures of muscle fatigue using EMG have shown conflicting results under low-level contraction conditions [6,7,9]. In general, at least 15% MVC is required to detect reliable changes in EMG signals caused by fatigue [12,13].

Measuring muscle twitch responses, on the other hand, provides an alternative solution to identify fatigue-related muscle state changes [10,14]. A muscle twitch response is a localized involuntary muscle contraction [15,16] that can be evoked using artificial muscle stimulation technology [17,18]. By using muscle stimulation, the corresponding muscle fatigue can be quantified as a significant reduction in measured stimulation responses comparing to pre-fatigue conditions [17,18,19,20]. Unlike the traditional EMG-based method, fatigue detected using muscle stimulation methods is not affected by the muscle contraction level. The muscle stimulation method has been validated as a method to measure potential muscle fatigue [18] and has been applied to various body segments, such as forearm muscles [14,21,22] or the lower back [10,23,24]. In the above studies, muscle forces or joint torques generated by muscle stimulation were used as a quantitative measure of muscle stimulation response. However, an accurate measurement of evoked muscle forces or torques relies highly on the accessibility of target muscles using force and torque measurement devices, e.g., a load cell or force gauge. In other words, the stimulation responses from the target muscles need to be accessible and measurable. For example, the flexor digitorum superficialis muscle in the forearm can be stimulated independently to generate isolated and measurable torque as a measure of the stimulation response [14,22]. However, no isolated and accessible muscle force or torque can be measured directly from some muscles, such as the shoulder mid-deltoid muscle, to quantify the corresponding twitch response.

To solve such potential issues, a computer vision-based approach can be used to quantify the stimulation responses of targeted muscles without being restricted by the abovementioned weaknesses. Computer vision provides a noninvasive solution to acquire information virtually. Computer vision has been used in object detection and tracking [25], classification [26], pattern recognition [27], etc. Computer vision technology can be used to detect human macroscopic movement [28,29] or subtle and even invisible microscopic movement, such as breathing activity [30] or facial pulse [31]. Computer vision is also widely used to quantify human physiological states, such as tracking eye blinking rate or facial expression for fatigue detection and distraction [32,33]. Considering their broad developments and applications, computer vision technologies can potentially work as a noninvasive technique to determine muscle contractile properties caused by artificial muscle stimulation through the measurement of visually acquirable information, e.g., the muscle belly displacement. The changes within measured displacements can be further linked to the development of muscle fatigue.

Therefore, the goal of this research was to develop a reliable computer vision-based method to quantify potential muscle fatigue resulting from prolonged low-load exposure. In this study, a computer vision-based method was developed to detect and track mid-deltoid muscle displacement to understand the fatigue development from low-load exposure over a prolonged period. We hypothesized that muscle fatigue developed during low-load prolonged exposure would be associated with reduced stimulation-evoked muscle belly displacement measured by the developed computer vision method.

## 2. Materials and Methods

To develop an effective computer vision-based method for quantifying stimulation response, the human deltoid muscle was chosen in this study. The deltoid muscle is usually considered to have three distinguishable anatomical sections, i.e., anterior, middle, and posterior sections, which can be further divided into seven muscle fascicle groups [34]. Each muscle section plays an essential role in the gross movement and stabilization of the arm [35]. The middle deltoid muscle contains the highest proportion of slow-twitch fibers (i.e., 39%) compared to the anterior deltoid (33%) muscle and the posterior deltoid (17%) muscle [36], which allow the middle deltoid muscle to provide continuous support against sustained low-load conditions. However, a single deltoid section is not powerful enough to drive the upper arm to complete an isolated movement. Therefore, it is challenging to quantify stimulation response and evaluate muscle fatigue development from individual deltoid sections using standard stimulation measurement methods as described above. The abovementioned characteristics make the middle deltoid muscle a perfect target to be studied; therefore, it was selected in this study to develop effective and reliable vision-based methods to capture the stimulation responses for the purpose of muscle fatigue detection.

### 2.1. Participant

A sample of six participants (three males and three females) completed the experiment. All participants gave informed consent prior to any data collection, following the procedures approved by the local Institutional Review Board. This study was approved by the local Institutional Review Board with the confirmation number HUM00090843. Their mean (SD) age, height, body mass, and body mass index (BMI) were 26.5 (3.2) years, 173.5 (5.7) cm, 65.5 (18.2) kg, and 22.6 (2.1), respectively. Inclusion criteria required that participants were physically active and had no illnesses, injuries, or musculoskeletal disorders within the past year that limited their daily activities. Individuals with body mass index >30 were also excluded due to the potential difficulty in evoking reliable muscle contractions.

### 2.2. Apparatus and Task

The left shoulder mid-deltoid muscle was selected to stimulate using a dual-channel, current-controlled muscle stimulator (Grass S88, AstroMed, Inc., West Warwick, RI, USA) in series with a stimulus isolation unit (SIU5, AstroMed, Inc., West Warwick, RI, USA) and a constant current unit (CCU1, AstroMed, Inc., West Warwick, RI, USA).

**Figure 1:**
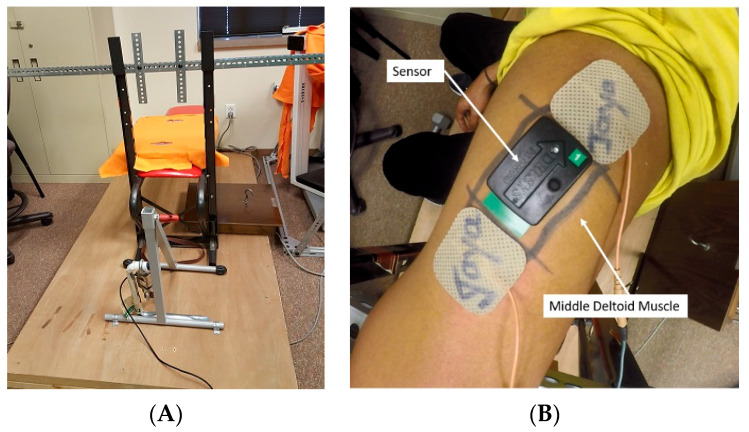
Illustration of the experimental setup: (**A**) experiment fixture; (**B**) stimulation electrode and EMG sensor locations.

During the experiment, participants sat in an experimental fixture (Figure 1A), in which the motion of the upper arm and shoulder was restrained. Participants were positioned in a comfortable and relaxed upright sitting posture. A pair of 3.4 cm × 5.1 cm bipolar stimulating electrodes (PALS^®^ Platinum Model NC89201, Axelgaard Manufacturing Co. Ltd., Fallbrook, CA, USA) were placed over the mid-deltoid muscle (Figure 1B). The location of the pair of the electrodes was derived from existing studies [37,38,39]. The electrode locations were marked above and below the center of the middle deltoid muscle at its origin and insertion positions. Stimulation responses from the muscle stimulation were sampled using a high-resolution motion camera (GoPro, Inc., San Mateo, CA, USA) at 60 frames per second under 2560 × 1440 pixels. The motion camera, mounted on top of a left metal bar on a fixture, was pointing at the stimulation area at roughly −85° from the horizontal direction verified using a goniometer. If participants consciously or subconsciously contracted their muscle voluntarily during the stimulation, the measured stimulation response could become inaccurate due to the contribution of the extra voluntary contraction. Therefore, to minimize such potential influence, voluntary muscle activities were continuously monitored using a bipolar wireless EMG electrode placed over the center of the mid-deltoid belly (Figure 1B). Muscle stimulation was started only after the observed level of muscle activities was (qualitatively) minimized without substantial perturbation in measured EMG levels.

### 2.3. Stimulation Protocol

**Figure 2:**
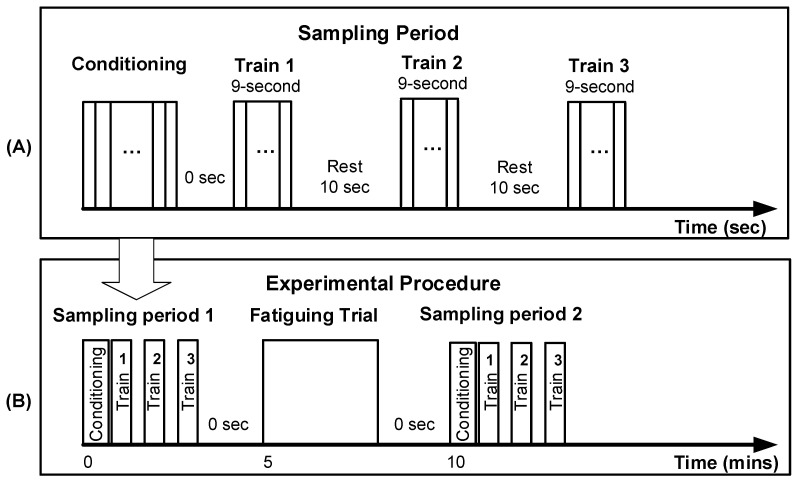
Illustrations of the overall experimental procedure: (**A**) muscle stimulation measurement protocol during one sampling period; (**B**) entire experimental procedure. No rest period was provided between the stimulation sampling period and fatiguing trial.

The participant’s skin around the mid-deltoid muscle region was first shaved, then cleaned with 70% alcohol isopropyl, and dried following published procedures [40]. A preparatory procedure was then completed to identify the most effective stimulation site for each individual according to the procedures described in previous studies [10,18]. The stimulation intensity and stimulation protocol were then determined following the procedure described in a previous study [10]. In general, stimulus intensity was set at 85% of the participant’s tolerance level. The stimulation protocol started with a muscle conditioning trial at 2 Hz to remove the potential measurement offset associated with twitch potentiation [19,41]. Right after the conditioning train, three 9 s stimulation trains at 2 Hz were applied with a 10 s break between each train (Figure 2A). The same stimulation trial was repeated before and after one fatiguing trial, as shown in Figure 2B. Here, 2 Hz was selected to deliver a stable and reliable stimulation response, as described in a previous study [10].

### 2.4. Experimental Procedure

In order to minimize potential influences from other physical activities, all participants were instructed not to engage in any intense physical activities 1 day before and on the day of the scheduled experiment session. At the beginning of data collection, participants were placed in the customized fixture (Figure 1B), and an initial stimulation protocol was then completed as described above. The sitting posture and arm position were marked on the fixture to minimize the potential repositioning effects. After completing the first stimulation session, participants were asked to complete a fatiguing trial. During the fatiguing trial, participants were required to holding their shoulder in a lateral abducted position until they could not hold such a posture anymore. Right after the fatiguing trial, participants were placed in the customized fixture again at the marked position, and the same stimulation protocol was then repeated on the participants. During both stimulation procedures, the stimulation responses were captured using the action camera described earlier. The room temperature and humidity were also centrally controlled at a constant level to minimize the potential impact on muscle conditions. As shown in Figure 2B, the entire experimental session took about 20 min to complete, which included about 10 min for the initial preparation, e.g., sensor attachment and skin preparation, and 12 min for the subsequent formal data collection and fatiguing trial.

### 2.5. Data Process and Analysis

**Figure 3:**
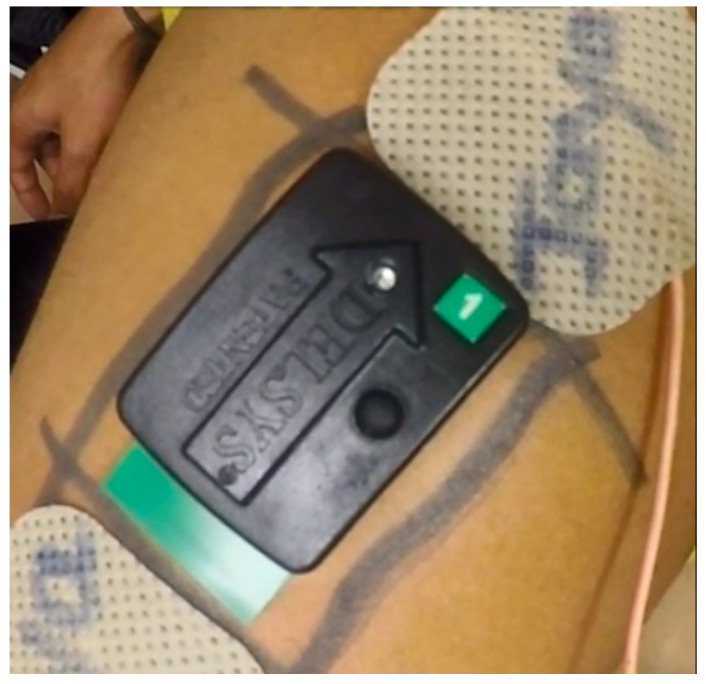
Illustration of region of interest (RoI) containing EMG sensor attached to the skin.

In this study, muscle displacements and changes caused by the stimulation were recorded using the camera as a measure of stimulation response. In order to capture the stimulation-caused muscle volume changes, the displacements of the center of the mid-deltoid muscle belly were selected to be studied using a computer vision-based method. To identify corresponding displacements, a region of interest (RoI) containing the target feature points was first identified from the camera’s field of view, as shown in Figure 2B. The area centered on the EMG sensor was selected as the RoI in this study, as shown in Figure 3. Many factors could potentially affect the magnitude and direction of the measure of RoI displacements, such as affinal distortion and 3D view position changes, which could introduce significant noise to later feature analysis and comparison. Each video was manually checked, whereby a stable video consisted of the EMG sensor approximately centered in the captured area for most of the time, while any unusual camera or subject hand movement was considered noise and cropped from the video. Therefore, in order to control for potential noise, only the stable RoI video length was considered for analysis. The profile information of each recorded video, including the filename, duration in seconds, frame rate, video resolution, video format, and the total number of frames, was obtained using Python (v3.9.6, Python Software Foundation, Wilmington, DE, USA) on an Intel Core i7-7700HQ CPU utilizing the OpenCV-python 4.5.1.48 library.

**Figure 4:**
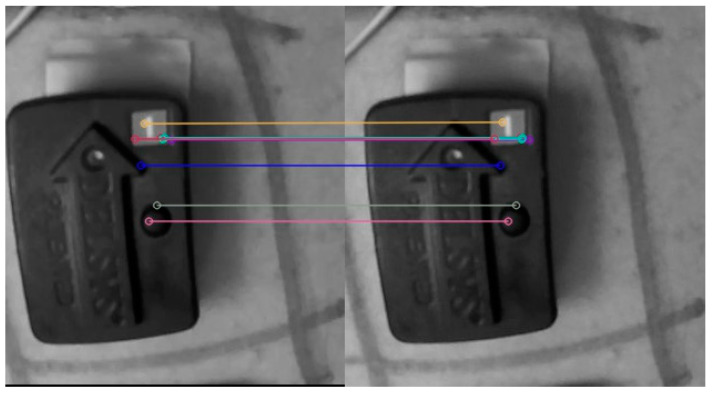
Illustrations of the same feature point in two adjacent frames and the corresponding displacement of the feature points between two adjacent frames.

The displacements of the matched features between adjacent frames were obtained through the following procedure: first, typical targeted features, such as lines, edges, circular spots, and corners, were determined to track changes of RoI from extracted frames. Then, the Oriented FAST and Rotated BRIEF (ORB) detector was used to detect, extract, and describe pixels from a frame [42]. Oriented FAST (Oriented Features from Accelerated Segment Test) is a corner detection method used to classify a pixel as a feature point [43]. A circle containing the target pixel p and 16 surrounding pixels was used for the estimation. Selected pixel p was classified as a feature point when more than eight pixels from the circle were brighter or darker than pixel p at each time frame. Then, each classified feature point was assigned a rating based on the number of pixels darker or brighter in the circle. A feature point received the maximum rating when all 16 pixels were brighter or darker, whereas the minimum rating was given when only nine pixels were brighter or darker. The top 10 rated feature points were shortlisted using the Harris corner method from a frame [44]. Each of the shortlisted feature points was described using the Rotated BRIEF (Rotated Binary Robust Independent Elementary Feature) method as encoded information such as pixel intensity, rotation angle, and neighboring pixel points [45]. For all shortlisted features, a descriptor was defined that consisted of a neighboring square patch around the feature point. These descriptors were converted into a binary vector that stores encoded information about the feature in numerical format. The rotated BRIEF method takes pixels from the neighborhood of each feature point to store pixel intensity and rotation angle in binary vector as a description. The top 10 features along with the descriptions from each of the extracted frames were stored in a multidimensional array. Furthermore, a similarity score between two adjacent frames was calculated using the matched features from the subsequent feature matching step. The Multi-Probe LSH (Multi-Probe Locality Sensitivity Hashing) method was used to conduct a similarity search, wherein features with a similar description having high probability from two adjacent frames were matched [46]. The BF Matcher (Brut Force Matcher) function was then used to calculate the distance between matched features using Hamming distance as the measurement of the displacement [47]. Figure 4 illustrates the matched features between two adjacent frames and the translation between them. These displacements of detected and matched features were extracted from each adjacent frame for the entire video to estimate the related stimulation response displacement and to further understand the fatigue development caused by the fatiguing trial.

In order to detect potential muscle fatigue, displacements measured from 10 s of video from before and after fatiguing trials were compared, and the corresponding changes were determined to quantify the potential fatigue effects. Here, muscle fatigue was detected if the overall magnitudes of displacements measured after the fatiguing trial were smaller than those measured before the fatiguing trial.

## 3. Results

**Figure 5:**
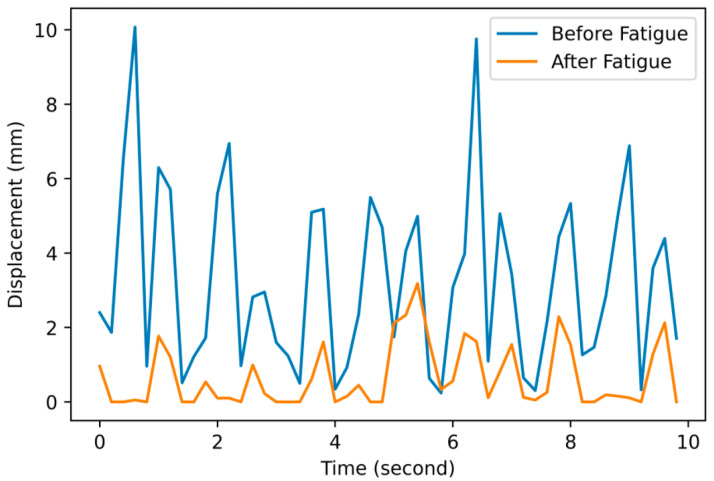
Illustration of detected stimulation responses (displacement) before and after fatiguing trial.

In summary, the mid-deltoid muscles were successfully evoked by the stimulation protocol, and corresponding stimulation responses were successfully estimated by capturing the RoI feature displacements, which were further used to detect potential muscle fatigue caused by the fatiguing trial. For each participant, feature displacement from each stimulation pulse was successfully captured using the developed vision-based method. The changes in the displacements before and after the fatiguing trial were further identified. In general, consistent drops were observed from all the measured displacements from 10 s of video, as illustrated in Figure 5.

**Figure 6:**
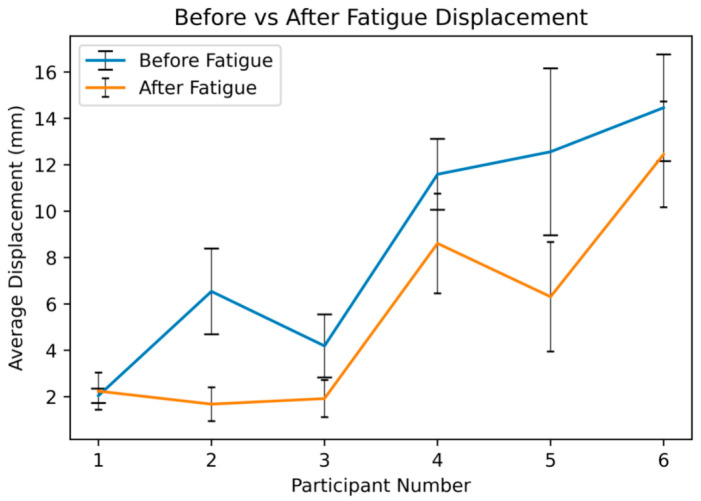
Average feature displacement detected during two muscle stimulation trials (i.e., before and after fatiguing trial).

As shown in Figure 6, the mean (SD) of muscle belly displacements before and after the fatiguing trial were 8.56 (0.99) mm and 5.53 (0.74) mm, respectively. Captured muscle displacements ranged from 1.56 to 18.77 mm before the fatiguing trial, and such a range decreased to 0.44 to 16.66 mm after the fatiguing trial. It can be inferred from the above summary in Figure 6 that there was a clear drop in average muscle displacement after the fatiguing trial, although the level of the drop in measured displacements was not consistent across all the participants. The average difference between before and after the fatigue stimulation response was about 3.03 mm across all participants.

## 4. Discussion

The objective of this research was to develop a reliable computer vision-based stimulation method to quantify potential muscle fatigue. A muscle stimulation protocol was applied to generate artificial muscle contraction before and after a fatiguing trial, and a computer vision-based algorithm was developed to track the muscle stimulation response, which was quantified by measuring the changes of targeted features caused by the muscle stimulation.

In summary, muscle stimulation responses were successfully quantified by measuring RoI feature displacements from collected video data across all six participants. From both before and after fatiguing trials, measured feature displacement data were analyzed to understand potential fatigue development resulting from the fatiguing trial. If the target muscle is fatigued after the fatiguing trial, the same amount of artificial electrical stimulation applied to the target muscle cannot cause the same amount of muscle contraction, which can be observed by the degree of changes from the observable surface muscle twitches. Thus, compared to data collected from the first stimulation session before fatiguing trial, a decreased amplitude of measured displacement was observed from the stimulation session after fatiguing trial across all six participants. On average, the observed reduction in displacement in before and after the fatigue stimulation response was 3.03 mm, and consistent drops in measured displacements across the entire 10 s of videos were observed in all six participants. Gorelick and Brown [37] also used the muscle stimulation method to study the deltoid muscle contractile properties. They used an MMG (mechanomyography) laser sensor to detect the stimulation response, and their results showed similar displacements captured by the MMG sensor. Overall, the results in this study are larger than in their study by several millimeters, which could have been caused by the fact that the stimulation settings applied in this study were stronger than in their study, and that the muscle evoked was the entire deltoid muscle rather than each deltoid segment. Since there are very few existing studies that measure shoulder muscle fatigue using similar methods, this study fills this critical gap by introducing a noninvasive method to measure the development of muscle fatigue.

The developed algorithm successfully captured the targeted RoI features continuously from all recorded videos samples, and the developed method was also sensitive to capture potential changes. As shown in Figure 6, participant 5 yielded the largest average displacement differences before and after the fatiguing trial, while participant 1 yielded the smallest average displacement changes. These results indicate that the same muscle stimulation protocol could lead to different muscle responses due to individual differences, which could be clearly reflected in the measured displacement changes. Although the magnitude was small, the proposed method captured the small changes in measured displacements consistently through each recorded video for all participants.

In addition to observed results, some limitations should be acknowledged. Firstly, only six participants were recruited for this study. A small sample size can potentially introduce large variations, which may affect the overall results. However, in general, the developed method provided reliable results across all tested participants. Even though large between-subject variations were observed, the developed method was able to deliver reliable and stable outcomes. Future study is needed to test the method across a larger population with higher diversity to further test its robustness and reliability. Secondly, the seated test position was not precisely controlled between the two sampling periods. Even though the sitting posture from the first sampling period was marked, there was still a chance to mislocate the participants during the second sampling period, which could have potentially introduced variability in measured stimulation responses. Even though different methods were applied to reduce the potential impacts of participant reposition, a certain level of impact was still expected. Future study is needed to improve the accuracy and robustness of the data collection sessions. Thirdly, voluntary contraction from connected muscles was quantitatively monitored using EMG sensors. No quantified EMG value was used to precisely control the level of voluntary muscle contraction. However, such an influence should be limited due to the data collections only starting when there was no observable disturbance in the monitored EMG, and the corresponding data were disregarded when any noticeable voluntary muscle contractions were observed during the stimulation period. Lastly, the camera was attached to the experimental fixture on which participants were asked to rest their shoulder and arm during the data collection. The muscle contraction caused by the stimulation occasionally caused overall movement of the experimental fixture.

## 5. Conclusions

In conclusion, this study proposed a computer vision-based method to capture musculature status changes caused by artificial stimulation. The results from this study can be used to further developed a computer vision-based muscle stimulation method to detect long-lasting muscle fatigue caused by low but consistent loading conditions, such as prolonged sitting.

## Data Availability

The data used to support the findings of this study are available upon request by contacting the corresponding author.
